# Three different mutations in the DNA topoisomerase 1B in *Leishmania infantum* contribute to resistance to antitumor drug topotecan

**DOI:** 10.1186/s13071-021-04947-4

**Published:** 2021-08-28

**Authors:** Chloé Rosa-Teijeiro, Victoria Wagner, Audrey Corbeil, Ilda d’Annessa, Philippe Leprohon, Rubens L. do Monte-Neto, Christopher Fernandez-Prada

**Affiliations:** 1 Département de Pathologie et Microbiologie, Faculté de Médecine Vétérinaire, Université de Montréal, Saint-Hyacinthe, QC Canada; 2 The Research Group on Infectious Diseases in Production Animals (GREMIP), Faculté de Médecine Vétérinaire, Université de Montréal, Saint-Hyacinthe, QC Canada; 3 Medtronic EMEA, Study and Scientific Solutions, Milan, Italy; 4 Centre de Recherche en Infectiologie du Centre de Recherche du Centre Hospitalier Universitaire de Québec, Université Laval, Quebec City, Canada; 5 Instituto René Rachou-Fundação Oswaldo Cruz/Fiocruz Minas, Belo Horizonte, Brazil; 6 Department of Microbiology and Immunology, Faculty of Medicine, McGill University, Montréal, QC Canada

**Keywords:** Protozoan parasites, *Leishmania*, DNA topoisomerases, Topotecan, Drug resistance, SNPs

## Abstract

**Background:**

The evolution of drug resistance is one of the biggest challenges in leishmaniasis and has prompted the need for new antileishmanial drugs. Repurposing of approved drugs is a faster and very attractive strategy that is gaining supporters worldwide. Different anticancer topoisomerase 1B (TOP1B) inhibitors have shown strong antileishmanial activity and promising selective indices, supporting the potential repurposing of these drugs. However, cancer cells and *Leishmania* share the ability to become rapidly resistant. The aim of this study was to complete a whole-genome exploration of the effects caused by exposure to topotecan in order to highlight the potential mechanisms deployed by *Leishmania* to favor its survival in the presence of a TOP1B inhibitor.

**Methods:**

We used a combination of stepwise drug resistance selection, whole-genome sequencing, functional validation, and theoretical approaches to explore the propensity of and potential mechanisms deployed by three independent clones of *L. infantum* to resist the action of TOP1B inhibitor topotecan.

**Results:**

We demonstrated that *L. infantum* is capable of becoming resistant to high concentrations of topotecan without impaired growth ability. No gene deletions or amplifications were identified from the next-generation sequencing data in any of the three resistant lines, ruling out the overexpression of efflux pumps as the preferred mechanism of topotecan resistance. We identified three different mutations in the large subunit of the leishmanial TOP1B (Top1B^*F187Y*^, Top1B^*G191A*^, and Top1B^*W232R*^). Overexpression of these mutated alleles in the wild-type background led to high levels of resistance to topotecan. Computational molecular dynamics simulations, in both covalent and non-covalent complexes, showed that these mutations have an effect on the arrangement of the catalytic pentad and on the interaction of these residues with surrounding amino acids and DNA. This altered architecture of the binding pocket results in decreased persistence of topotecan in the ternary complex.

**Conclusions:**

This work helps elucidate the previously unclear potential mechanisms of topotecan resistance in *Leishmania* by mutations in the large subunit of TOP1B and provides a valuable clue for the design of improved inhibitors to combat resistance in both leishmaniasis and cancer. Our data highlights the importance of including drug resistance evaluation in drug discovery cascades.

**Graphical abstract:**

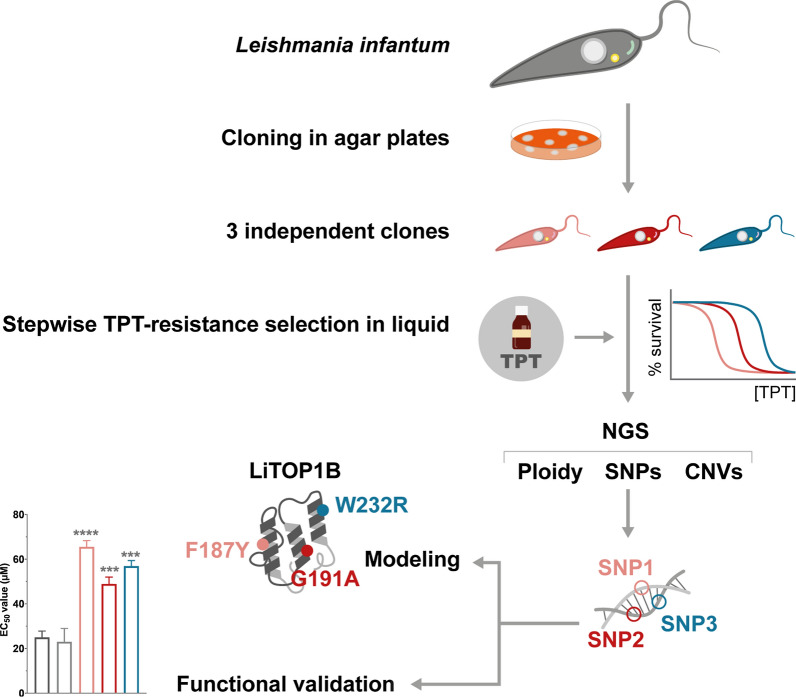

**Supplementary Information:**

The online version contains supplementary material available at 10.1186/s13071-021-04947-4.

## Background

Despite 1 million new cases of leishmaniasis being declared every year, there is still no effective vaccine available for humans. Moreover, available treatments for this major neglected tropical disease (NTD) are limited and outdated [1]. Clinical manifestations vary in severity and include cutaneous, mucocutaneous, and visceral leishmaniasis. The latter form, caused by *Leishmania infantum*, is fatal within 2 years if left untreated [2]. Leishmaniasis is also responsible for a significant health, psychosocial, and economic burden around the world [3, 4]. Due to the limited pharmacopeia, most available antileishmanial drugs are used in both humans and dogs. Of note, dogs constitute the main reservoir for the zoonotic life cycle of *Leishmania* [5, 6].

The rapid emergence and spread of resistant parasite strains has prompted the need for new intervention pathways and antileishmanial drugs. In this way, repurposing of approved drugs has become a very attractive strategy to tackle leishmaniases and is gaining supporters worldwide [1, 7, 8]. DNA topoisomerases (TOP) have garnered attention since their discovery by James Wang in 1971 [9]. TOP are key enzymes for many essential biological functions such as DNA replication, transcription, recombination, DNA repair, and DNA segregation [10]. TOP enzymes are preferential targets against rapid-dividing, highly proliferative eukaryotic cells, such as those responsible for various types of cancer (e.g. ovarian cancer or metastatic carcinoma of the colon) [11, 12]. Type 1B TOP (TOP1B) are ATP-independent enzymes that relax topological tensions in supercoiled DNA during replication and transcription processes mediated by DNA and RNA polymerases. TOP1B enzymes produce topological changes (e.g. relaxation) in supercoiled DNA substrates through a five-step coordinated sequence: (i) DNA binding; (ii) DNA cleavage and formation of a transient protein–DNA covalent interaction; (iii) DNA controlled rotation to release torsional stress; (iv) DNA religation; and (v) DNA release. Eukaryotic TOP1B are monomeric enzymes, except in kinetoplastid organisms (e.g. *Leishmania*) that rely on heterodimeric TOP1B enzymes, with coding genes located in different chromosomes, 34 and 4, respectively [13, 14]. The major structural and functional differences between *Homo sapiens* TOP1B (hTOP1B) and *L. infantum* TOP1B (LiTOP1B) [15, 16], coupled with *Leishmania*’s rapid proliferation rate, offer great potential for selective chemotherapy [13]. Different anticancer TOP1B inhibitors, including the water-soluble camptothecin derivative topotecan (TPT) currently used to treat ovarian carcinoma [11], have been successfully tested against *Leishmania* parasites in in vitro*, *ex vivo, and in vivo murine models, demonstrating strong antileishmanial activity and promising selective indices, supporting the potential repurposing of these drugs [8, 17, 18]. However, tumor cells and *Leishmania* parasites share an undesirable and important feature in being prone to becoming resistant to drug treatment. *L. infantum* relies on DNA copy number variations (CNVs) for regulating the expression of drug targets [19, 20]) and drug resistance genes [20, 21]. In addition to CNVs, single-nucleotide polymorphisms (SNPs) in drug target genes or in transporters can lead to drug resistance without the need for altering gene content [22–24]. Markedly, recent studies point to the need for experimental generation of drug resistance to promising compounds in order to clinically evaluate and eventually circumvent the phenomenon [25–27].

The goal of this study was to complete a whole-genome exploration of the effects caused by prolonged exposure to TPT to highlight the different potential drug resistance mechanisms (changes in ploidy, CNVs, SNPs, etc.) deployed by *L. infantum* to favor its survival in the presence of an FDA-approved TOP1B inhibitor. This information is critical to the fight against drug-resistant *Leishmania* parasites, furthering knowledge of the mechanisms these parasites use to persist in the presence of drugs, as well as serving as a foundation for improved drug repurposing strategies.

## Methods

### *Leishmania* cultures

*Leishmania infantum* (MHOM/MA/67/ITMAP-263) wild-type (WT) promastigotes and mutants resistant to > 700 μM TPT (TPT700.1, TPT700.2, and TPT700.3) generated in vitro in a stepwise manner were grown in M199 medium at 25 °C supplemented with 10% fetal bovine serum, and 5 μg/ml of hemin at pH 7.0. In addition, 700 μM of TPT (topotecan hydrochloride hydrate, Sigma-Aldrich) was added to the media for the maintenance of the endpoint mutants. Growth curves were performed in 25 cm^2^ cell culture flasks by seeding 1 × 10^6^ parasites/ml, and the number of parasites was determined daily—up to 7 days—by manual counting using the Neubauer hemocytometer. Growth assays were performed with at least three biological replicates from independent cultures (*n* = 3), each of which included three technical replicates.

### Mutant selection

Three *L. infantum* WT independent clones, which were obtained by plating the MHOM/MA/67/ITMAP-263 strain onto solid M199, were independently selected in 25 cm^2^ flasks containing 5 ml M199 medium supplemented with 10% heat-inactivated fetal bovine serum (FBS) and 5 μg/ml hemin in the presence of increasing TPT concentrations, as described previously [23]. Briefly, the stepwise drug selection ranged from 1 × the EC_50_ of TPT (24 μM as determined in the present study) up to 16 × the EC_50_ of TPT (384 μM), with a twofold increase in drug concentration (24, 48, 96, 192, and 384 μM) every three sub-culturing passages. Topotecan hydrochloride hydrate (Sigma-Aldrich, St. Louis, MO, USA) was used as the source of TPT.

### Drug susceptibility assays

Antileishmanial values in promastigotes were determined by monitoring the growth of parasites after 72 h of incubation at 25 °C in the presence of increasing concentrations of TPT, by measuring A_600_ using a Cytation 5 multimode reader (BioTek, USA). Drug efficacy assays were performed with at least three biological replicates from independent cultures (*n* = 3). EC_50_ values were calculated based on dose–response curves analyzed by nonlinear regression with GraphPad Prism 8.4.3 software (GraphPad Software, La Jolla, CA, USA). Statistical analyses were performed using unpaired two-tailed *t*-tests. A *P*-value < 0.05 was considered statistically significant.

### Whole-genome sequencing of TPT-resistant mutants

Comparative whole-genome sequencing (WGS) was performed as described previously [23]. Briefly, genomic DNA was prepared from a mid-log phase clonal culture of each TPT-resistant mutant. DNA was quantified fluorometrically, and 50 ng of material was used for library preparation using a Nextera™ DNA Sample Preparation Kit (Illumina) according to the manufacturer’s instructions. The size distribution of Nextera™ libraries was validated using an Agilent 2100 Bioanalyzer and High Sensitivity DNA chips (Agilent Technologies). Sequencing libraries were quantified with the QuantiFluor^®^ dsDNA System and sequenced using an Illumina MiSeq platform with 250-nucleotide paired-ends reads. An average genome coverage of over 50-fold was obtained for the mutants. This approach allowed identification of SNPs when compared with the reference genome sequence of *L. infantum* JPCM5 (TriTrypDB v9.0) [28] and *L. infantum* 263 WT [29]. Sequence reads were aligned to the *L. infantum* JPCM5 genome and *L. infantum* 263 WT using the software bwa-mem [30]. The maximum number of mismatches was four, the seed length was 32, and two mismatches were allowed within the seed. Read duplicates were marked using Picard (http://broadinstitute.github.io/picard), and GATK was applied for InDel realignment and SNP and InDel discovery in the three TPT mutants. PCR amplification and conventional DNA sequencing verified SNPs of interest detected by WGS. CNVs were derived from read depth coverage by comparing the coverage of uniquely mapped reads between each of the three TPT mutants and the *L. infantum* 263 WT in 5 kb non-overlapping genomic windows for the 36 chromosomes (normalized to the total number of uniquely mapped reads for each strain) [31]. The sequence data for the *L. infantum* TPT-resistant mutants is available at the NCBI BioProject (https://www.ncbi.nlm.nih.gov/bioproject/) under study accession PRJNA647847 and sample accessions SAMN15599759, SAMN15599760, and SAMN15599761, corresponding to TPT-resistant clones TPT700.1, TPT700.2, and TPT700.3, respectively.

### Quantitative real-time RT-PCR

RNAs from the WT and the three mutants were extracted using the RNeasy Mini Kit (Qiagen) according to the manufacturer's recommendations. The cDNA was synthesized using the iScript™ Reverse Transcription Supermix (Bio-Rad), and amplified in the iTaq™ universal SYBR^®^ Green Supermix Kit (Bio-Rad) using a CFX Opus Real-Time PCR System (Bio-Rad). The expression levels of *LinJ.34.3220* (Fw: 5′-CGACTTCGAGCCCATTTATCA-3′; Rv: 5′-ACCTTCAGCTTGCCCATTAG-3′) and *LinJ.04.0070* (Fw: 5′-CCCTCCGTCAAGAAAGTTGT-3′; Rv: 5′-CTCTTCCACATTGCCCAGT-3′) were derived from three technical and three biological replicates and were normalized to constitutively expressed mRNA encoding glyceraldehyde-3-phosphate dehydrogenase (*LinJ.36.2480*; Fw: 5′-GTACACGGTGGAGGCTGTG-3′; Rv: 5′-CCCTTGATGTGGCCCTCGG-3′).

### DNA constructs and nucleofection

The WT allele of *LinJ.34.3220* (DNA topoisomerase IB large subunit) and its three mutant variants were amplified from genomic DNA derived from either *L. infantum* or mutants TPT700.1, TPT700.2, and TPT700.3 (mutations T560A, G572C, and T724C, respectively) using compatible primer pairs (Fw: 5′-*Xba*I-ATGAAGGTGGAGAA-3′; Rv: 5′-*Hind*III-TACACCCTCAAAGC-3′). PCR fragments were ligated into a pGEM T-easy vector (Promega) to confirm the quality of the insert by Sanger sequencing. PCR fragments were then cloned in the *Leishmania* expression vector pSP72α*hyg*α, which contains the gene hygromycin phosphotransferase (*hyg*), a selectable marker in *Leishmania* [32]. A total of 20 μg of plasmid DNA for episomal expression, either the empty vector (mock) or carrying the genes of interest, were delivered into *L. infantum* WT promastigotes by nucleofection, as described previously [33]. Selection was achieved in the presence of a final concentration of 300 μg/ml hygromycin.

### Computational methods for the in silico study of *Leishmania* topoisomerase 1B

The three-dimensional structure of the *L. donovani* TOP1LS complex (PDB ID: 2B9S [34]) contains residues 27–456 and 221–262 of the large and small subunits, respectively. Residues missing in the crystal structure, 427–430 of the large subunit, have been modelled as reported in Roy et al. [35]. Following this procedure, a non-covalent interaction of the LiTOP1 bi-subunit with a 22-base-pair DNA substrate was modelled using the program UCSF Chimera [36] by fitting the structure of the protein backbone atoms on the coordinates of the human enzyme trapped in complex with the DNA (PDB ID 1A36). The same procedure was carried out in order to model an LiTOP1-DNA covalent interaction, using the human TOP1-DNA-topotecan ternary complex, extrapolated from the PDB structure 1K4T [37].

Once the two systems (wild-type LiTOP1–DNA covalent and non-covalent complexes) were obtained, the program UCSF Chimera [36] was used to introduce the three point mutations identified in our whole-genome sequencing experiments (F187Y, G191A, and W242R). All systems were subjected to molecular dynamics (MD) simulations aimed at understanding the structural/dynamical effect of the mutations, thus explaining their impact on TPT sensitivity. Briefly, each covalent and non-covalent complex was placed in a triclinic simulative box filled with a water molecule TIP3P model [38], and the resulting systems were rendered electroneutral by the addition of sodium counterions; this step, i.e. the topology building, was performed using the Amber 14 all-atoms force field, highly suitable for simulating nucleic acids [39]. The systems were first subjected to a round of minimization of 10,000 steps of steepest descent followed by 10,000 steps of conjugate gradient. Relaxation of water molecules and thermalization in NPT environment were carried out for 1.2 ns in time steps of 1 fs. Six runs of 200 ps each were carried out while increasing the temperature by 50 K at each step, ranging from 50 to 300 K.

The systems were then simulated with a 2 fs time step for 300 ns in periodic boundary conditions, using a cut-off of 8 Å for the evaluation of short-range non-bonded interactions and the particle mesh Ewald method for long-range electrostatic interactions [40]. The temperature was kept constant at 300 K with Langevin dynamics [41], whereas pressure was fixed at 1 atmosphere through the Langevin piston method [42]. The bond lengths of solute and water molecules were restrained with the SHAKE [43] and SETTLE [44] algorithms, respectively. Atomic positions were saved every 250 steps (i.e. 0.5 ps) for analysis with the Gromacs 4.6 package [45].

## Results

### In vitro resistance selection and characterization of the TPT-resistant mutants

**Fig. 1:**
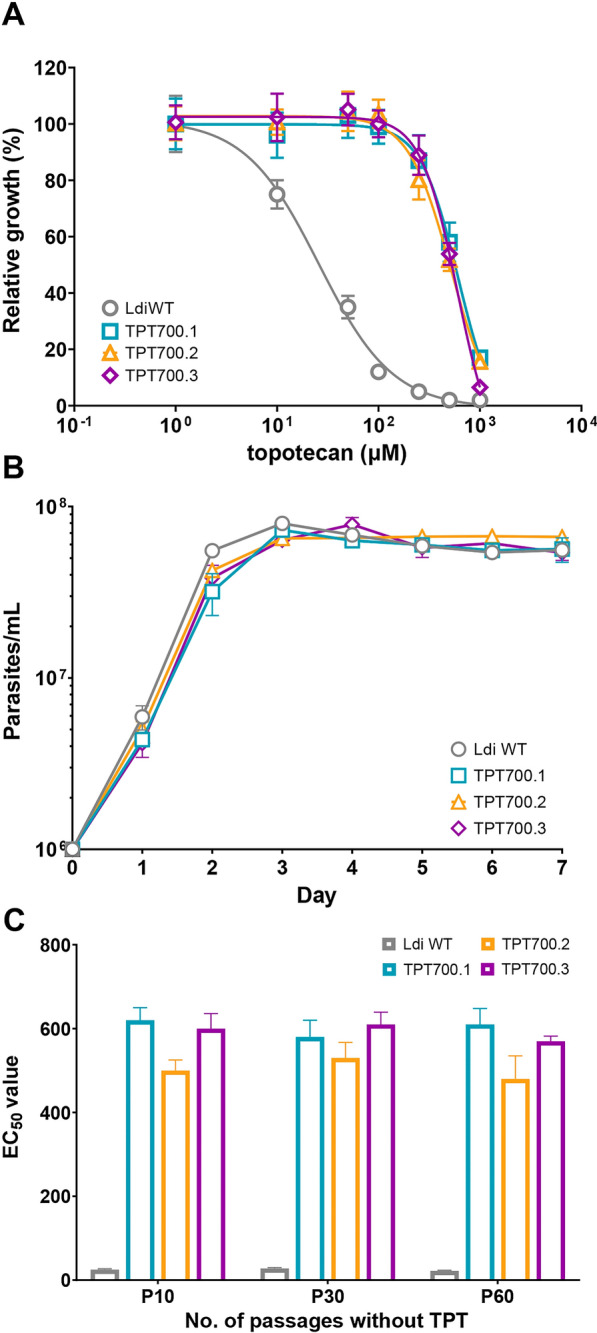
Phenotypic characterization of *L. infantum* TPT-resistant mutants selected in vitro. **a** Concentration–response curves for *L. infantum* WT and TPT-resistant promastigotes in the presence of growing concentrations of TPT. EC_50_ values were calculated from concentration–response curves performed in triplicate after nonlinear fitting with GraphPad Prism 8.4.3 software: 25, 612, 519, and 602 µM for the WT, TPT700.1, TPT700.2, and TPT700.3, respectively. **b** Growth profiles of *L. infantum* WT and the three TPT-resistant mutants after reaching the last selection step. Parasites were seeded in M199 medium at a final concentration of 10^6^ parasites ml^−1^, and their growth was monitored daily for 7 days by manual counting. For **a** and **b**, data are the mean ± SEM of three biological replicates. Statistical analyses were performed using unpaired two-tailed *t*-tests. **c** EC_50_ calculation for the three TPT-resistant mutants after 10, 30, and 60 passages without TPT in the cell culture media

Selection for resistance to TPT was performed to evaluate the possible propensity for drug resistance to this anticancer TOP1B inhibitor if repurposed against visceral leishmaniasis. Experimental concentration–response assays with *L. infantum* WT promastigotes revealed a TPT-sensitive phenotype within the low micromolar range (Fig. 1a; EC_50_ = 25 µM). Selection of independent TPT-resistant mutants began at 1 × EC_50_ up to 16 × EC_50_ of the WT parental line. The selection procedure was fast (three subsequent passages per drug concentration), and cells rapidly adapted to growing concentrations of TPT. All three clones reached the final selection step at 16 × EC_50_ (passage 3). At this point, clones were re-evaluated in terms of growth ability and sensitivity to TPT (Fig. 1a). All three clones were able to survive concentrations higher than 700 µM (ca. 28 × EC_50_ of the WT), demonstrating very high EC_50_ values (612, 519, and 602 µM for TPT700.1, TPT700.2, and TPT700.3, respectively) compared to the parental WT strain (~ 25 µM). Once the final selection step was reached, the growth rate of each TPT mutant was determined in order to verify their fitness compared to the WT parent line. As depicted in Fig. 1b, the TPT-resistant phenotype experimentally induced in promastigotes did not lead to any significant difference in the growth rate of the parasite when compared to the WT cells (Fig. 1b). Resistant phenotypes were stable after growth in media lacking TPT for over 60 passages (Fig. 1c).

### Comparative WGS

Whole-genome sequencing was performed to compare the three independent TPT-resistant strains to the WT line to pinpoint and further explore potential mechanisms underlying the resistant phenotype. WGS was conducted by Illumina next-generation sequencing on the three independent *L. infantum* TPT700-resistant lines selected at 16 × EC_50_, as well as the isogenic *L. infantum* WT counterpart line. For all strains, this produced genome assemblies of 31 Mb with a coverage depth of at least 50-fold.

**Fig. 2:**
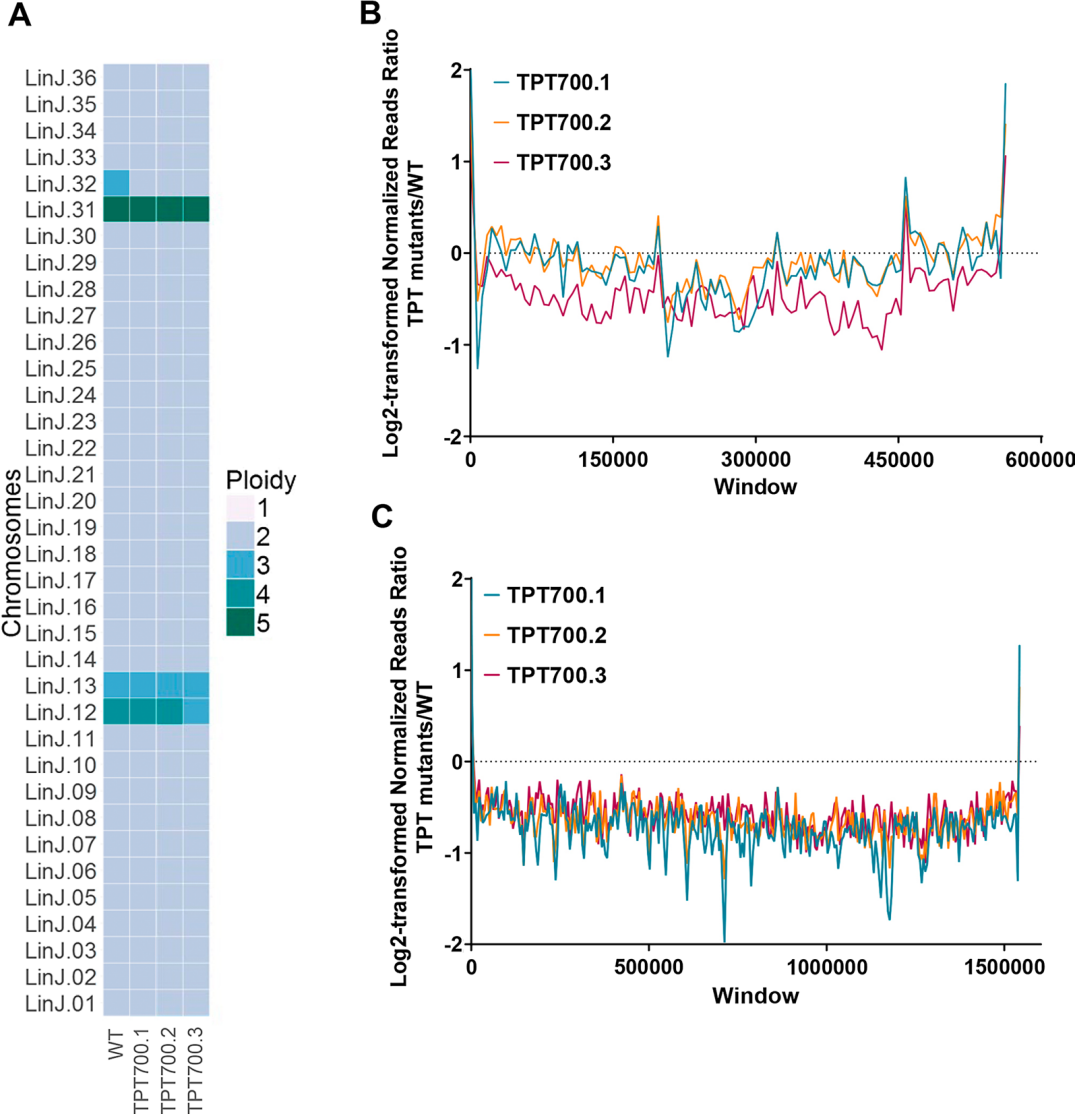
Dynamics of aneuploidy of *L. infantum* after in vitro adaptation to TPT.** a** Heatmap representation of log_2_-transformed normalized TPT-resistant/WT read ratio for all 36 chromosomes in the three independent *L. infantum* TPT-resistant lines selected at 16 × EC_50_. Chromosomes were divided into non-overlapping 5 kb genomic windows, and the median *L. infantum* TPT-resistant/WT read ratios for each chromosome were normalized according to the total number of reads followed by log_2_ transformation. **b** Log_2_-transformed 16 × TPT-resistant mutants/WT reads ratios for non-overlapping 5 kb genomic windows on chromosome 12. **c** Log_2_-transformed 16 × TPT-resistant mutants/WT reads ratios for non-overlapping 5 kb genomic windows on chromosome 32

No significant deletions or amplifications were identified; however, the TPT-resistant lines demonstrated aneuploidy compared to the WT parental line. Two cases of reduction in ploidy were observed in the TPT-resistant mutants (Fig. 2a; Additional file 1: Dataset S1). These losses affected chromosome 12 in clone TPT700.3 (Fig. 2a, b) and chromosome 32 in the three resistant mutants (Fig. 2c). The log_2_ TPT700.3/WT read ratio for chromosome 12 (Fig. 2b) was close to −0.5, which should represent a loss of one allele compared to WT parasites (going from four to three chromosome copies). In the same way, the log_2_ TPT/WT read ratio (Fig. 2c) for chromosome 32 was close to −1, pinpointing a trisomic-to-disomic shift in all three TPT-resistant mutants (Fig. 2c), which could correlate with reduced TPT sensitivity. No cases of supernumerary chromosomes were observed in the TPT-resistant mutants (Fig. 2a; Additional file 1: Dataset S1).

A search for point mutations revealed several homozygous and heterozygous SNPs in the different TPT-resistant mutants (Additional file 2: Dataset S2). Nineteen common genes were mutated in the three TPT-resistant mutants (Table 1). In order to find potential SNP candidates, the different clones were examined for SNPs occurring in the same ORF but at different positions (to avoid mutations that are most likely to occur as natural polymorphisms in the parental strain). SNPs fulfilling these characteristics occurred in eight genes, including two coding for hypothetical proteins (*LinJ.19.1680* and *LinJ.19.1690*), three proteophosphoglycan-coding genes (*LinJ.35.0490*, *LinJ.35.0510*, and *LinJ.35.0550*) often found mutated in our various sequencing screens, *LinJ.24.1120* (encoding a putative pre-mRNA splicing factor), *LinJ.34.0710* (encoding a putative flagellar attachment zone protein), and the gene coding for the large subunit of the heterodimeric LiTOP1B (*LinJ.34.3220*). The three different mutations identified in the LiTOP1B ORF by next-generation sequencing (NGS) and confirmed by targeted PCR were homozygous mutations *top1B*^*F187Y*^ and *top1B*^*W232R*^ for TPT700.1 and TPT700.3 mutants, respectively, and heterozygous SNP *top1B*^*G191A*^ for TPT700.2. The levels of *top1B* (*LinJ.34.3220* and *LinJ.04.0070*) mRNA did not vary between the different clones and the WT parental cell line (Additional file 3: Table S1).

**Table 1:** Overview of the common mutated genes in the three TPT-resistant mutants

Mutant	Gene ID	Mutation position	Allele frequency	Ref/Mut AA	Gene function
TPT700.1 TPT700.2 TPT700.3	*LinJ.01.0420*	1073 1073 1073	Het Het Het	A/E A/E A/E	Hypothetical protein conserved
TPT700.1 TPT700.2 TPT700.3	*LinJ.09.0680*	1388 1388 1388	Hom Hom Hom	na na na	Hypothetical protein conserved
TPT700.1 TPT700.2 TPT700.3	*LinJ.09.0950*	17 17 17	Hom Hom Hom	na na na	Polyubiquitin
TPT700.1 TPT700.2 TPT700.3	*LinJ.12.0662*	425 425 425	Het Het Het	S/T S/T S/T	Surface antigen protein putative
TPT700.1 TPT700.2 TPT700.3	*LinJ.19.1680*	3792 3665 2940	Het Het Hom	na V/A E/D	Hypothetical protein
TPT700.1 TPT700.2 TPT700.3	*LinJ.19.1690*	3956 3389 3956	Het Het Het	S/N G/A S/N	Hypothetical protein
TPT700.1 TPT700.2 TPT700.3	*LinJ.24.1120*	771 776 771	Het Het Het	S/R S/F S/R	Pre-mRNA splicing factor putative
TPT700.1 TPT700.2 TPT700.3	*LinJ.27.0870*	2572 2572 2572	Het Het Het	R/G R/G R/G	Hypothetical protein conserved
TPT700.1 TPT700.2 TPT700.3	*LinJ.28.2320*	856 856 856	Hom Hom Hom	N/Y N/Y N/Y	Hypothetical protein conserved
TPT700.1 TPT700.2 TPT700.3	*LinJ.28.2390*	1321 1321 1321	Hom Hom Hom	na na na	Cyclin-dependent kinase-binding protein putative
TPT700.1 TPT700.2 TPT700.3	*LinJ.28.2450*	3758 3758 3758	Hom Hom Hom	na na na	DNA topoisomerase ii
TPT700.1 TPT700.2 TPT700.3	*LinJ.29.1450*	242 242 242	Hom Het Hom	A/V A/V A/V	Amastin-like protein
TPT700.1 TPT700.2 TPT700.3	*LinJ.29.2240*	1267 1267 1267	Hom Hom Hom	L/F L/F L/F	Hypothetical protein conserved
TPT700.1 TPT700.2 TPT700.3	*LinJ.31.1470*	1088 1088 1109	Hom Hom Het	na na na	Hypothetical protein unknown function
TPT700.1 TPT700.2 TPT700.3	*LinJ.34.0710*	2129 76 2843	Het Het Het	G/D A/P D/G	Flagellar attachment zone protein putative
TPT700.1 TPT700.2 TPT700.3	*LinJ.34.3220*	560 572 724	Hom Het Hom	F/Y G/A W/R	DNA topoisomerase IB large subunit
TPT700.1 TPT700.2 TPT700.3	*LinJ.35.0490*	16,451 1992 16,285	Hom Het Hom	R/H na V/I	Proteophosphoglycan ppg4
TPT700.1 TPT700.2 TPT700.3	*LinJ.35.0510*	7549 5278 2437	Het Het Het	A/T V/L G/S	Proteophosphoglycan ppg4
TPT700.1 TPT700.2 TPT700.3	*LinJ.35.0550*	1699 2471 2471	Het Het Hom	A/T na na	Proteophosphoglycan ppg1

The information depicting all reference/mutant nucleotide changes is contained in Additional file 2: Dataset S2

*Het *heterozygous, *Hom* homozygous, *na* not applicable due to frameshift

### Impact of LiTOP1B mutant variants in *L. infantum* TPT drug resistance

**Fig. 3:**
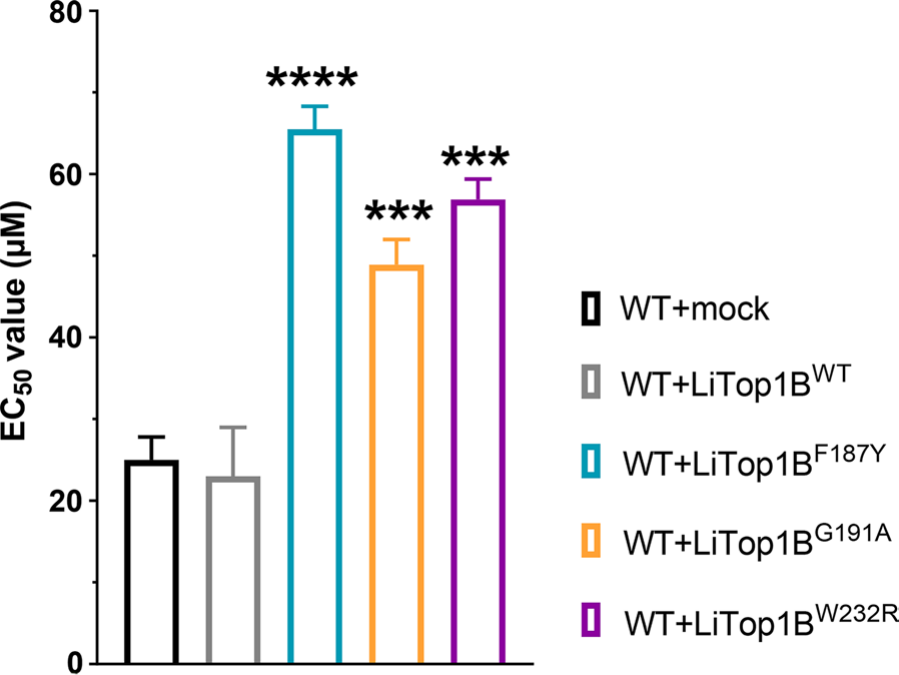
Impact of overexpression of the leishmanial *top1B*^*WT*^ gene, as well as three mutated versions, on the TPT sensitivity profile of *L. infantum*. EC_50_ values were calculated from concentration–response curves performed in triplicate after nonlinear fitting. Data are the mean ± SEM of three biological replicates. Differences were statistically evaluated by unpaired two-tailed *t*-test (***P ≤ 0.001, ****P ≤ 0.0001)

Because *top1B* is an essential gene in *Leishmania*, our preferred method for studying the role of TOP1B mutations was episomal transfection of WT and mutated forms of the gene in the WT background (Fig. 3). To this end, the WT copy as well as the three mutated forms of *top1B* were recovered by PCR, confirmed by Sanger sequencing, and episomally overexpressed in the WT background. Markedly, while episomal overexpression of gene *LinJ.34.3220* (WT or mutated) increases the potential amount of TOP1B large subunit available, the amount of functional TOP1B heterodimer in the cell remains stable (independent of the overexpression), as it is limited by the basal amount of TOP1B small subunit. An increase in the total amount of functional WT TOP1B heterodimer in the presence of TPT would lead to an increase in the sensitivity to TPT. Overexpression of the leishmanial *top1B*^*WT*^ allele did not confer TPT sensitivity in the WT cell line when compared with the mock control, confirming that the amount of TOP1B heterodimer remains stable. On the other hand, if the SNPs found in the mutated variants of the TOPIB large subunit play a role in TPT resistance and are able to bind to the small subunit (competing with the WT form), these would lead to a reduced sensitivity towards TPT. As depicted in Fig. 3, overexpression of all three mutated variants of the *top1B* gene resulted in a significant decrease in TPT sensitivity in the WT background: *top1B*^*F187Y*^ (2.62-fold; *P* < 0.0001), *top1B*^*G191A*^ (1.96-fold; *P* = 0.0006), and *top1B*^*W232R*^ (2.28-fold; *P* = 0.0001).

### Structural effect of the SNPs and impact on TPT sensitivity

The mechanism of inhibition exerted by TPT against TOP1B is strictly dependent on the enzyme catalytic cycle. Once the DNA cleavage has occurred, TPT is able to intercalate the DNA bases and stabilize the protein–DNA covalent complex, thus blocking the cycle and stalling the enzyme. If one of the catalytic steps is compromised, this can affect TPT sensitivity.

**Fig. 4:**
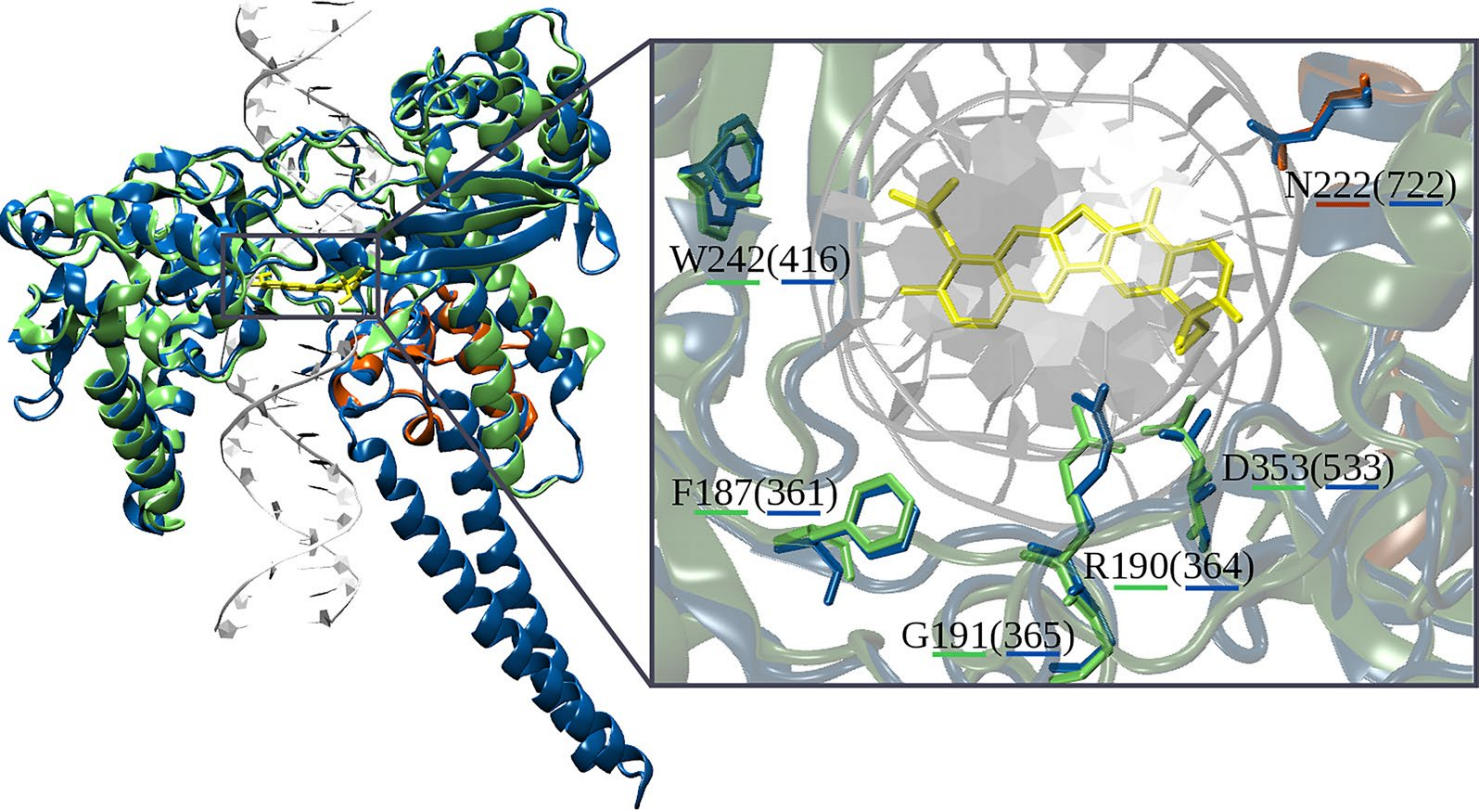
Structural alignment between LiTop1B (PDB 2b9s) and hTop1B crystallized in covalent complex with DNA and in the presence of the inhibitor topotecan (PDB 1k4t). The large and small subunits from the dimeric structure of LiTOP1B are shown in green and orange, respectively, while hTop1B is reported in light blue, and topotecan is shown in yellow. The insert panel shows topotecan together with the structural alignment of residues forming the binding pocket (hR364, hD533, and hN722) and the residues of LiTOP1B that, if mutated, lead to drug resistance (LiF187, LiG191, and LiW242)

In order to evaluate the impact of the point mutations on the structure of the protein and its interaction with the DNA substrate for a correct progression of the catalytic cycle, we performed molecular dynamics (MD) simulations of the WT and mutant enzymes (F187Y, W232R, and G191A). In particular, we carried out MD simulations both of the WT and mutant non-covalent and covalent TOP1B–DNA complexes (for a total of eight systems), allowing detection of the mutations’ effect in any step of the two phases of the catalytic cycle, i.e. cleavage and religation. This gives important insight into the mechanism(s) underlying resistance due to the fact that these mutations are located in close proximity to the catalytic pentad and to the TPT binding site (Fig. 4).

**Fig. 5:**
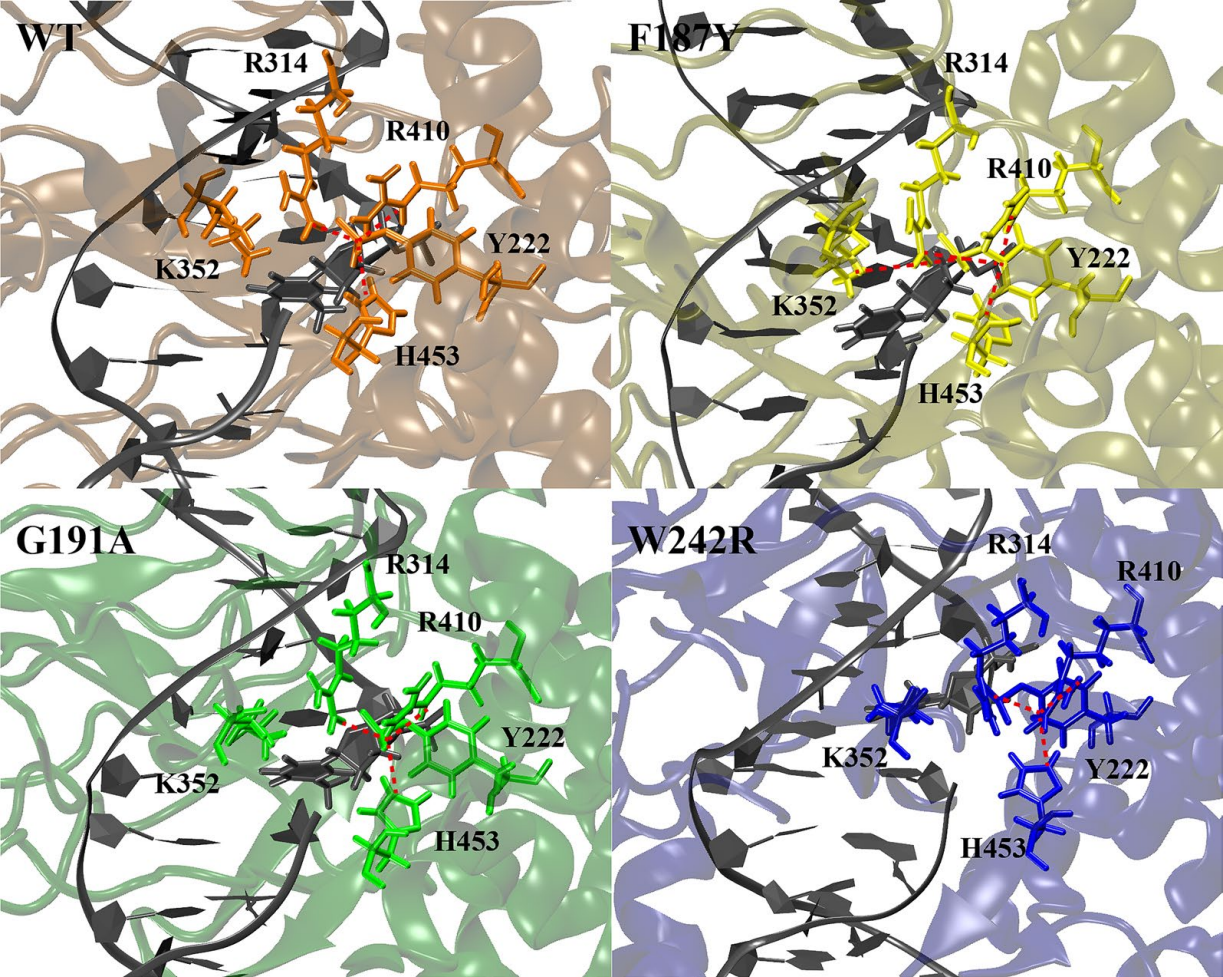
Covalent simulations reporting the three-dimensional arrangement of the catalytic pentad in the four systems and its intra-pentad and pentad-DNA hydrogen bonds. WT: top-left panel; F187Y: top-right panel; G191A: bottom-left panel; and W242R: bottom-right panel. The residue–residue interactions are highlighted by a red line

**Fig. 6:**
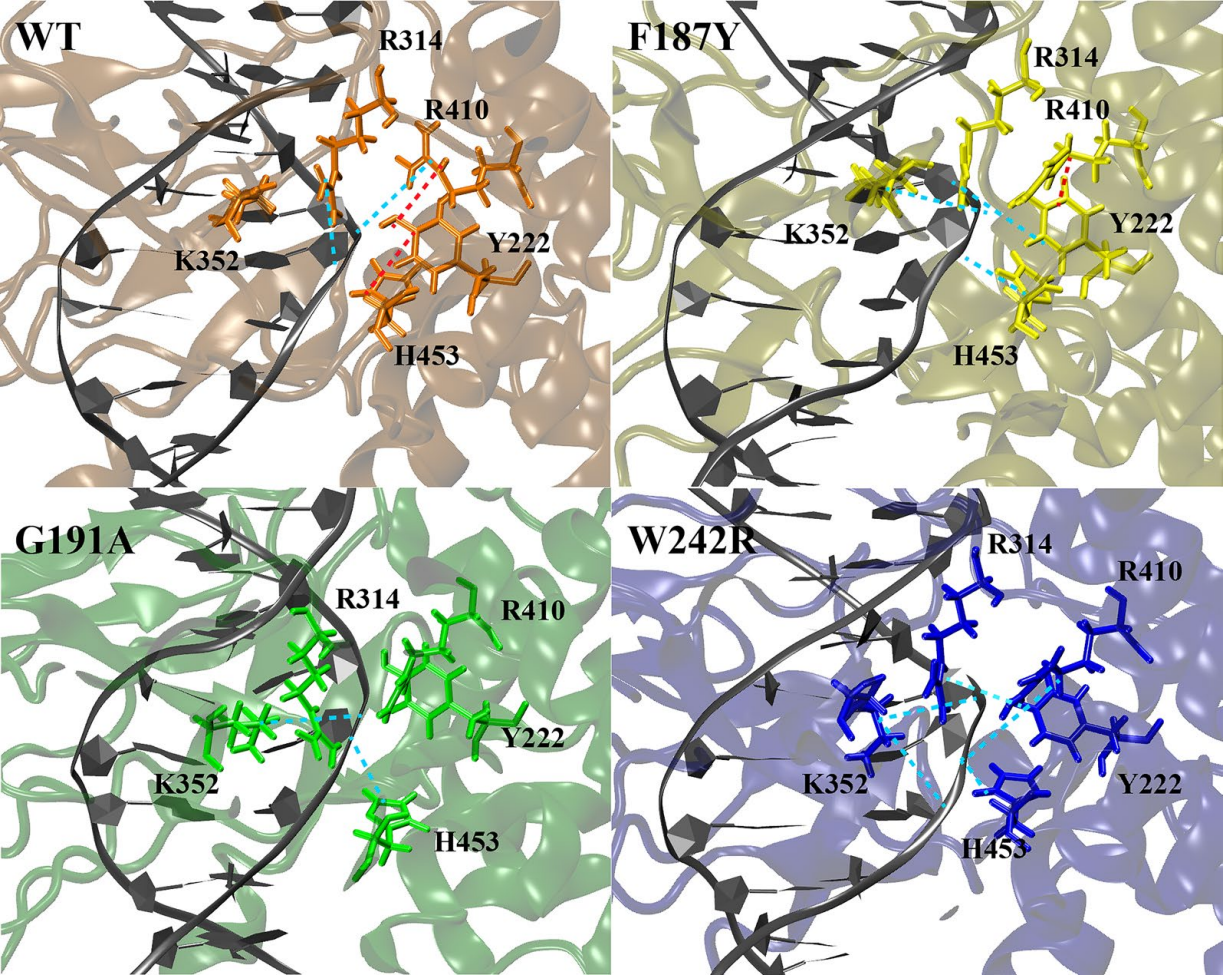
Non-covalent simulations, reporting the three-dimensional arrangement of the catalytic pentad in the four systems and its intra-pentad and pentad-DNA hydrogen bonds. WT: top-left panel; F187Y: top-right panel; G191A: bottom-left panel; and W242R: bottom-right panel. The residue–residue interactions are highlighted by a red line, while those occurring with the DNA are shown with a light blue line

In both groups of simulations (covalent and non-covalent complexes), the mutation of each residue has an effect on the arrangement of the catalytic pentad (R314, K352, R410, and H453 of the large subunit and Y222 of the small subunit) (Figs. 5, 6) and on the interaction of these residues with the surrounding amino acids and the DNA substrate (Tables 2, 3). This likely alters the cleavage/religation equilibrium, which is at the basis of an altered drug sensitivity. Of note, the profile of interaction at the cleavage site (−1/+1 bases) of the protein–DNA in the non-covalent complex is hardly affected in all mutants, making us hypothesize that due to the improper arrangement of the catalytic pentad, the reaction rate is affected. Similarly, the catalytic pentad arrangement is impacted, in particular at the level of residue K352, which is crucial for the religation reaction to proceed and for the stabilization of TPT in the DNA binding pocket.

**Table 2:** Analysis of the hydrogen bond network in covalent complex simulations

	WT	F187Y	G191A	W232R
Active site pentad and surrounding residues				
ARG314:PTR222s	97.78	98.26	97.97	98.82
CYS329:ARG314	98.46	98.33	98.38	98.12
LYS352:ASP353	60.41			
ILE355:LYS352	78.65	77.83	78.79	86.33
ARG410:ALA406	44.55			
ARG410:PTR222s	99.72	99.79	99.67	99.55
ARG410:ALA312	85.33	97.66	91.83	97.50
ARG410:CYS451		41.72		48.01
ALA414:ARG410	90.37	90.21	85.86	89.70
HIS453:ALA202s	85.22	82.20	82.39	80.23
HIS453:PTR222s	97.64	90.28	96.79	90.86
SER214s:HIS453	93.57	93.87	87.36	92.65
PTR222s:SER218s	76.09	65.74	66.94	67.68
Residues of point mutations and surrounding residues				
PHE(Y)187:LYS198	99.05	99.37	99.40	99.53
GLY189:PHE(Y)187		80.85	69.95	64.26
GLY197:PHE187	93.63	99.31	99.53	99.56
HIS193:GLY(A)191		65.35		65.47
TRP(R)242:LYS225	96.20	84.25	98.36	95.50

Percentage of the hydrogen bonds established by the active site pentad with the surrounding residues (PTR stands for phosphotyrosine, while “s” following the residue number indicates that residue belongs to the small subunit)

**Table 3:** Analysis of the hydrogen bond network in non-covalent complex simulations

	WT	F187Y	G191A	W232R
Active site pentad and surrounding residues and DNA bases				
ARG314:ASN452			69.42	
CYS329: ARG314	98.76	97.96	98.37	97.98
LYS352:ASP353	70.96	68.41	88.26	
ILE355:LYS352	57.14	73.93	96.42	75.04
ARG410:ALA406	44.32	61.96	97.86	
ARG410:ALA312	90.58	98.60	75.04	90.59
ARG410:TYR222s	59.80	83.62		
ARG410:CYS451		45.97		
ALA414:ARG410	89.56	89.25	96.27	90.98
HIS453:ALA202s	79.59	85.58	90.06	86.04
SER214s:HIS453	85.85	93.02	76.47	88.41
TYR222s:SER218s	68.45	73.21	78.77	64.46
ARG314:THY +1	87.60	98.50	73.82	88.02
LYS352:THY −1		52.21	68.58	53.88
LYS352:THY +1				74.12
ARG410:THY +1	75.18	86.34		
HIS453:THY +1	94.79	96.96	55.49	87.59
TYR222s:THY −1	65.22	80.60		43.97
TYR222s:THY +1	55.33			70.77
Residues of point mutations and surrounding residues				
PHE(Y)187:LYS198	99.25	99.14	99.33	99.48
GLY189:PHE(Y)187				44.74
GLY197:PHE187	92.48	99.16	99.29	99.52
HIS193:GLY(A)191			46.89	61.69
TRP(R)242:LYS225	98.45	98.33	98.03	94.67
TRP(R)242:LYS182				73.40

Percentage of the hydrogen bonds established by the active site pentad with the surrounding residues (“s” following the residue number indicates that residue belongs to the small subunit)

## Discussion

Topotecan is a semi-synthetic, water-soluble analogue of camptothecin (CPT), as well as the first FDA-approved oral TOP1B inhibitor for the treatment of several types of cancer. CPT derivatives have been repeatedly suggested as a good source of repurposed drugs for the treatment of a variety of infectious diseases caused by protozoan parasites [1, 13, 46]. With the alarming decrease in effectiveness of first-line drugs in areas where *L. infantum* is endemic, repurposed drugs could represent a faster solution at lower cost [47]. However, the predisposition of *Leishmania* to develop drug resistance should be addressed when repurposing a drug [1, 25, 27]. In this study, we used a combination of stepwise drug resistance selection, whole-genome sequencing, and theoretical approaches to explore the propensity of and potential mechanisms deployed by three independent clones of *L. infantum* to resist the activity of the TOP1B inhibitor TPT. One of the major strengths of this approach is that both the parent cell line and three directly derived drug-resistant lines are studied together, and thus, any confounding factor derived from strain-related heterogeneity is excluded from the analysis [23, 48].

Firstly, we demonstrated that *L. infantum* is able to become resistant to high concentrations of TPT. While the mechanisms involved in TPT resistance have not been fully elucidated in tumor cells, several studies have shown the implication of different drug transporters, such as multidrug resistance-associated protein 1 (ABCC1/MRP1) or the ABCG2 transporter [49, 50]. Likewise, *Leishmania* parasites rely on the amplification of ABC transporter MRPA (ABCC) and inactivation of the aquaglyceroporin 1 (AQP1) gene to counter the action of antimonial drugs [21, 23, 51]. As gene expression in *Leishmania* is regulated predominantly by gene dosage [20, 21], we proceeded to search for large-scale copy number variations (deletions and duplications) in chromosomes of the three selected clones for comparison with the unselected parental line. In the past, different ABCG and ABCC efflux-pump gene clusters were identified in *L. infantum* as part of chromosomes 6, 23, and 31 [52]. In addition, overexpression (with no changes in gene dosage) of the ABCG6 transporter is known to be involved in CPT resistance in *Leishmania* parasites [53]. Of note, none of these regions was found amplified in any of the three TPT-resistant clones in our whole-genome comparative analysis.

The absence of significant amplifications, coupled with the very unusual fact that no prominent changes in ploidy were observed for any of the studied clones [54], led us to suspect the potential implication of SNPs and small nucleotide insertions or deletions (indels) in the TPT-resistant phenotypes. Several SNPs have been shown to contribute to drug resistance (e.g. miltefosine, antimonial drugs, etc.) in *Leishmania* parasites by altering the activity of specific transporters or modifying different detoxification pathways [22, 23, 29, 55, 56]. Here we focused on SNPs and indels present in the three TPT-resistant clones and, at the same time, occurring in the same ORF but at different positions. Among the eight genes fulfilling these criteria, we identified the ORF coding for the large subunit of the DNA topoisomerase IB, which is the main target of CPT derivatives once bound to the DNA during cell replication [10, 11]. Clones TPT700.1 and TPT700.3 displayed homozygous mutations in the *top1B* gene, while the SNP identified in TPT700.2 was heterozygous. Although previously observed in diploid *Leishmania* parasites, homozygous mutations are rare because of their “nonreversible” nature. The homozygous mutations in clones TPT700.1 and TPT700.3 may have originated from loss of heterozygosity, a well-described phenomenon in *Leishmania* [57, 58]. Importantly, these results reinforce previous works demonstrating the possibility that, although rare, *Leishmania* can generate SNPs associated with drug resistance without the need for alteration of its genomic architecture and gene expression [59].

Due to the impossibility of generating a null mutant, our preferred method for studying the role of mutated variants of *top1B* consisted of episomal transfection of the mutated forms into a WT strain [23]. Since these transfected parasites still carry the *top1B*^*WT*^ allele, the mutated, overexpressed forms of the protein are in competition with the WT large subunit in their binding to the small subunit (in order to make up the functional heterodimer). As such, we were able to only partly recreate the highly resistant phenotype observed in the TPT700 mutants. However, the relative strength of each mutation followed the same drug resistance trend in both the original mutants (TPT700.1 > TPT700.3 > TPT700.2) and the episomal transfectants (*top1B*^*F187Y*^ > *top1B*^*W232R*^ > *top1B*^*G191A*^).

To better understand the potential contribution of these three SNPs to TPT resistance in the mutants, we performed several MD simulations. All three residues identified in the TPT700 mutants (F187, G191, and W232) were conserved between the human and parasitic enzyme and can be structurally aligned (Fig. 4). They were located in proximity of the TPT binding site, in close proximity to residues found to be crucial in the human enzyme for the interaction and stabilization of TPT with residues R364 and N722 once intercalated between DNA bases [60], corresponding to leishmanial residues R190 and N221. Thus, we can hypothesize that a change in one of the residues of this cluster may influence the arrangement of the TPT binding site. Although these residues are in proximity of the catalytic pentad [11, 14, 61], the mutations identified in this study are likely affecting the ability of binding of TPT to the DNA-TOP1B complex without altering the global catalytic function of the enzyme. Since TPT hinders DNA rotation within the covalent complex, a reduced binding of this drug would result in a faster religation step. Indeed, it has been very well established that a malfunction of cleavage/religation reactions will be reflected through altered protein drug sensitivity [11, 60]. Of note, two of the three SNPs identified in the TPT700 mutants (F187Y and G191A) were located within the conserved region corresponding to amino acids 361–365 in the hTOP1B enzyme. These results confirm the findings of Rubin et al*.* showing that a substitution of residue F361 can induce high levels of resistance against a CPT derivate (e.g. 9-nitro-20(*S*)camptothecin) in human U-937 myeloid leukemia cells [62]. Likewise, Li et al*.* showed that certain substitutions in the 361–364 region affect DNA cleavage/ligation by the enzyme, as well as contribute to resistance against CPT since they may be included in the CPT-binding domain [63]. These results suggest that these mutations are able to modify the architecture of the binding site, decreasing the persistence of TPT in the binding pocket, as well indicate that CPT and TPT may share binding sites in the LiTOP1B–DNA complex.

Furthermore, in the covalent complexes, K352 and R410 demonstrate a changed profile of interaction in all three TPT700 mutants. In particular, the hydrogen bond between K352 and D353 is lost. This interaction is crucial for the correct position of K352 (known to be a key player in the religation reaction), and when incorrectly positioned affects the religation rate and thus TPT sensitivity [15, 64]. Moreover, D353 is itself involved in the network of residues and TPT interaction. As such, the lack of the K352-D353 hydrogen bond and side chains orientation may be a main cause for rearrangement of the TPT binding site and lowered stabilization of the drug in the binding pocket, further explaining the observed resistance.

Importantly, the three TPT700 mutants became resistant to TPT without impairing their ability to proliferate in vitro. The process of becoming drug-resistant can lead to different evolutionary disadvantages (“fitness cost”), such as reduced survival [65]. However, this concept remains controversial in *Leishmania* and is highly dependent on the parasite’s genetic and environmental context [48]. Likewise, drug-resistant cancer cell lines exhibit different fitness–cost profiles, including subpopulations with increased fitness when compared to their sensitive counterparts [66]. The absence of fitness cost in vitro in the TPT700 mutants could be due in part to the fact that these cells do not use ATP-dependent drug efflux pumps to resist treatment (e.g. MRPA in antimony resistance), for which they would have to divert energy away from proliferation towards running of the pumps. Moreover, the absence of a cost in terms of growth would also explain why the TPT700 mutants did not return to sensitivity once the TPT was withdrawn. Mutants of this type have the potential to become a major risk for the spread of drug resistance into an environment devoid of antileishmanial drugs. However, at this point, whether this phenomenon would be stable in vivo or in the vector remains to be evaluated.

## Conclusions

This study represents the first whole-genome characterization of *Leishmania* parasites repeatedly exposed to a TOP1B inhibitor. Unlike current antileishmanial agents, TPT resistance did not have a major impact on leishmanial genomic organization or TOP1B expression levels, and did not lead to changes in gene dosage of known genes coding for efflux pumps; a phenomenon previously reported for CPT analogues in cancer cells. Of note, we found that these mutations could decrease the binding of TPT to the DNA-TOP1B binary complex, as well as lower the stabilization of the drug in the binding pocket of the leishmanial heterodimeric enzyme. Altogether, this work helps elucidate the previously unclear potential mechanisms of TPT resistance in *Leishmania* by mutations in the large subunit of TOP1B, and provides a valuable clue for the design of improved inhibitors to combat drug resistance. Due to the conserved nature of the mutated amino acids, this knowledge could also provide important means of overcoming resistance to TOP1B-specific drugs and developing diagnostic tools to detect TPT-resistant tumor cells. Finally, this study supports and expands the importance of including drug resistance assessments in drug discovery and drug repurposing cascades before proposing a molecule as a potential prototype for treatment of parasitic diseases.

## Supplementary Information

**Additional file 1: Dataset S1.** Log_2_-transformed 16 × TPT-resistant mutants/WT reads ratios for non-overlapping 5 kb genomic windows for the 36 *L. infantum* chromosomes in the three TPT-resistant strains.

**Additional file 2: Dataset S2.** Homozygous and heterozygous SNPs in the different TPT-resistant mutants.

**Additional file 3: Table S1.***LinJ.34.3220 *(*top1B* large subunit) and *LinJ.04.0070* (*top1B* small subunit) RNA expression in *Leishmania* cells.

## Data Availability

All data generated or analyzed during this study are included in this published article and its additional files. The sequence data for *L. infantum* TPT-resistant mutants is available at the NCBI BioProject (https://www.ncbi.nlm.nih.gov/bioproject/) under study accession PRJNA647847 and sample accessions SAMN15599759, SAMN15599760, and SAMN15599761, corresponding to TPT-resistant clones TPT700.1, TPT700.2 and TPT700.3, respectively.

## Abbreviations

A_600_: Absorbance at 600 nm
ABC: ATP binding cassette
AQP1: Aquaglyceroporin 1
CNVs: DNA copy number variations
CPT: Camptothecin
dsDNA: Double-stranded DNA
EC_50_: Half maximal effective concentration
FDA: US Food and Drug Administration
*hyg*: Hygromycin phosphotransferase gene
hTOP1B: *Homo sapiens* DNA topoisomerase 1B
InDel: Insertion-deletion
LiTOP1B: *Leishmania donovani infantum* DNA topoisomerase 1B
MD: Molecular dynamics
MRP: Multidrug resistance-associated protein
NGS: Next-generation sequencing
NTD: Neglected tropical disease
ORF: Open reading frame
SNP: Single-nucleotide polymorphism
TOP: DNA topoisomerase
TOP1B: Type 1B DNA topoisomerase
TPT: Topotecan
WGS: Whole-genome sequencing
WT: Wild type
