# Supplementary material for: Three different mutations in the DNA topoisomerase 1B in Leishmania infantum contribute to resistance to antitumor drug topotecan
Source: Parasit Vectors. 2021 Aug 28;14:438. doi: 10.1186/s13071-021-04947-4 (PMC8399852; doi:10.1186/s13071-021-04947-4)
Supplement: Supplementary file 3 — Additional file 3: Table S1.LinJ.34.3220 (top1B large subunit) and LinJ.04.0070 (top1B small subunit) RNA expression in Leishmania cells. [file 13071_2021_4947_MOESM3_ESM.docx]

**Supplementary Table S1:** ***LinJ.34.3220* (*top1B* large subunit) and *LinJ.04.0070* (*top1B* small subunit) RNA expression in *Leishmania* cells.** *LinJ.34.3220* and *LinJ.04.0070* mRNA levels were analyzed by quantitative real-time RT-PCR. The RNA expression ratios were normalized to GAPDH expression.

| **mRNA fold change** | **WT** | **TPT700.1** | **TPT700.2** | **TPT700.3** |
| --- | --- | --- | --- | --- |
| *LinJ.34.3220* | 1.09 ± 0.18 | 1.16 ± 0.15 | 0.98 ± 0.21 | 1.12 ± 0.11 |
| *LinJ.04.0070* | 0.99 ± 0.10 | 1.09 ± 0.20 | 0.96 ± 0.15 | 1.18 ± 0.24 |
